# Growth differentiation factor 6 derived from mesenchymal stem/stromal cells reduces age-related functional deterioration in multiple tissues

**DOI:** 10.18632/aging.100982

**Published:** 2016-06-14

**Authors:** Daisuke Hisamatsu, Michiko Ohno-Oishi, Shiho Nakamura, Yo Mabuchi, Hayato Naka-Kaneda

**Affiliations:** ^1^ Laboratory for Stem Cell Competency, RIKEN Center for Integrative Medical Sciences (IMS), Tsurumi-ku, Yokohama, Kanagawa 230-0045, Japan; ^2^ Department of Physiology, Keio University School of Medicine, Shinjuku-ku, Tokyo 160-8582, Japan; ^3^ Department of Biochemistry and Biophysics, Graduate School of Health Care Sciences, Tokyo Medical and Dental University, Bunkyo-ku, Tokyo 113-8510, Japan

**Keywords:** mesenchymal stem cells, aging, miR-17, Gdf6, stem cell aging, SASP

## Abstract

The senescence-associated secretory phenotype (SASP) has attracted attention as a mechanism that connects cellular senescence to tissue dysfunction, and specific SASP factors have been identified as systemic pro-aging factors. However, little is known about the age-dependent changes in the secretory properties of stem cells. Young, but not old, mesenchymal stem/stromal cells (MSCs) are a well-known source of critical regenerative factors, but the identity of these factors remains elusive. In this study, we identified growth differentiation factor 6 (Gdf6; also known as Bmp13 and CDMP-2) as a regenerative factor secreted from young MSCs. The expression of specific secretory factors, including Gdf6, was regulated by the microRNA (miRNA) miR-17, whose expression declined with age. Upregulation of Gdf6 restored the osteogenic capacity of old MSCs in vitro and exerted positive effects in vivo on aging-associated pathologies such as reduced lymphopoiesis, insufficient muscle repair, reduced numbers of neural progenitors in the brain, and chronic inflammation. Our results suggest that manipulation of miRNA could enable control of the SASP, and that regenerative factors derived from certain types of young cells could be used to treat geriatric diseases.

## INTRODUCTION

Senescent cells express myriad inflammatory cytokines and chemokines, a phenomenon termed the senescence-associated secretory phenotype (SASP) [1-3]. The SASP connects cellular senescence with age-related decline in tissue function, and specific SASP proteins act as systemic factors to promote aging. For instance, age-dependent increases in the levels of the chemokine CCL11 [4] and β2-microglobulin [5] impair hippo-campal neurogenesis and cognitive function, and C1q-mediated activation of Wnt signaling impairs muscle repair by inducing dysfunction in skeletal muscle satellite cells [6]. Clearance of senescent cells delays the induction of various geriatric pathologies, supporting the notion that the SASP promotes aging in a non-cell-autonomous fashion [7, 8]. Although very few anti-aging functions of the SASP have been reported, the pathway has been shown to serve as a surveillance system for cellular senescence [1, 9]. For example, oncogene-induced senescent hepatocytes secrete SASP-like inflammatory cytokines that attract attention from immune cells. These pre-malignant senescent cells are usually eliminated by the CD4-positive (CD4+) T cell-mediated adaptive immune response, but impairment of this system by CD4+ T cell deficiency results in the development of hepatocellular carcinoma. However, little is known about the disruption of tissue homeostasis by the cellular SASP and its influence on aging at the organismal level.

Aging of adult tissue stem cells also contributes to loss of tissue homeostasis, e.g., dysregulation of hematopoiesis due to a decline in the lymphopoietic potential of hematopoietic stem/progenitor cells (HSCs) [10, 11]; reduced muscle repair capacity due to diminished activity of skeletal muscle satellite cells [12-14]; and loss of multipotency (i.e., the potential to differentiate into osteogenic, adipogenic, chondrogenic, neurogenic, and myogenic lineages) of mesenchymal stem/stromal cells (MSCs). Previous studies showed that regenerative efficacy is greater in young MSCs than in old MSCs [15-17]. Moreover, the expression of growth factors declines with age [18, 19], and secretory factors exert different effects on MSC function in young vs. old MSCs [16, 20]. These reports suggest that age-dependent changes in secretory properties are induced in MSCs, although to date the MSC SASP remains uncharacterized.

MSCs are a source of various humoral factors, including vascular endothelial growth factor (VEGF), insulin-like growth factor-1 (IGF-1), and hepatocyte growth factor (HGF) [21]. The effects of some of these factors have been determined. For instance, TNF-α-induced protein 6 (TSG-6), an anti-inflammatory protein secreted from transplanted MSCs, exerts a beneficial effect in the mouse model of myocardial infarction [22]. Combined administration of monocyte chemoattractant protein-1 (MCP-1) and the ectodomain of sialic acid-binding Ig-like lectin-9 (Siglec-9), which are both secreted from dental pulp-derived MSCs, induces an anti-inflammatory environment, promoting functional recovery after spinal cord injury [23]. However, the secretory factors responsible for the regenerative effects of transplanted MSCs remain largely unknown.

In this study, we identified growth differentiation factor 6 [Gdf6; also known as bone morphogenetic protein (Bmp)-13 and cartilage-derived morphogenetic protein (CDMP-2)] as a regenerative factor secreted by young MSCs. Administration of Gdf6 restored differentiation potential of aged MSCs *in vitro* and exerted beneficial effects on some age-associated pathologies in mice.

## RESULTS

### Characterization of the age-related phenotypes of old MSCs

**Figure 1:**
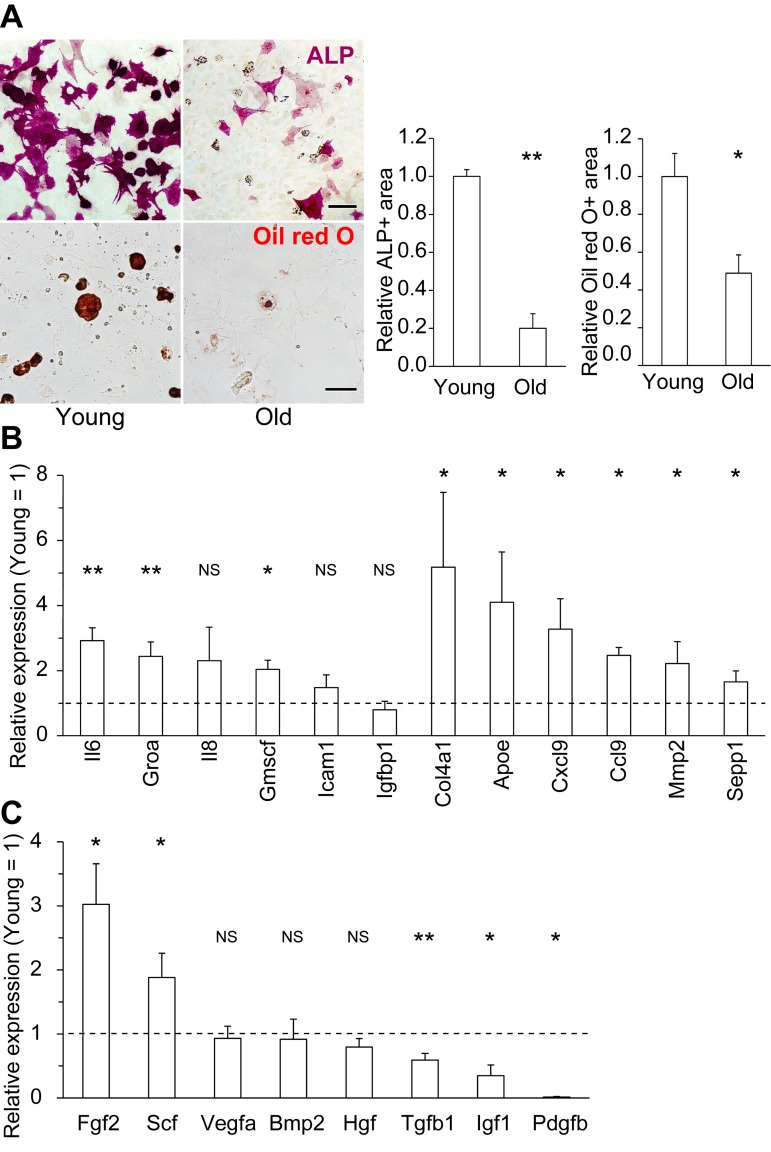
Changes in the differentiation potential and secretory profiles of old MSCs (**A**) Representative immunocytochemical images of young and old MSCs differentiated into ALP+ osteoblasts (upper) and Oil red O+ fat droplet-producing adipocytes (lower). Both the osteogenic and adipogenic potentials of old MSCs were reduced (n ≥ 3). (**B**) Expression levels of representative SASP factors (Il6, Groa, Il8, Gmscf, Icam1, and Igfbp1) and major secretory factors in old MSCs, relative to those in young MSCs, determined by qPCR (n ≥ 3). (**C**) qPCR of representative growth factors (n ≥ 3). Scale bars: 50 μm. Results are expressed as means ± standard error of the mean (SEM). NS (not significant), p > 0.05; *p < 0.05; **p < 0.01.

First, we compared the phenotypes of young (2–3 months) and old (> 18 months) MSCs. To exclude the influence of contaminating differentiated cells, such as lineage-restricted osteoblasts and adipocyte progenitors, we used fluorescence-activated cell sorting (FACS) to isolate MSC fractions (CD45-, CD31-, TER119-, Pdgfra+, and Sca-1+) from young and old murine bone marrow (BM) [24, 25]. We confirmed upregulation of the representative cellular senescence markers p21 (also known as Cdk1na) and senescence-associated β-galactosidase activity (SA-β-gal) (Supplementary Fig. 1). Consistent with previous reports, both differentiation potential and secretory profiles differed between young and old MSCs [19]. Specifically, the osteogenic and adipogenic potential of MSCs declined with age (Fig. 1A). In addition, old MSCs expressed higher levels of inflammatory factors, including representative SASP factors such as interleukin 6, Groa, and Gmcsf, and also exhibited alterations in the expression levels of certain growth factors (Fig. 1B–C).

### miR-17 overexpression restores the differentiation potential of old MSCs

To identify genes capable of restoring the regenerative capacity of old MSCs, we compared the gene expression profiles of young and old MSCs using microarrays and microRNA (miRNA) quantitative PCR (qPCR) arrays (Supplementary Tables 1 and 2). These analyses identified several candidate genes that were highly expressed in young MSCs and downregulated with age, including the miR-17 family members miR-17, -18a, -19a/b, -20a/b, -25, -93, -92a, -106a/b, and -363 (Supplementary Table 3). Previously, we showed that miR-17 and its paralogs miR-106a/b are responsible for the neurogenic differentiation potential of neural stem/progenitor cells (NSCs) during early developmental stages, and that overexpression of miR-17 restores the neuropotency of gliogenic NSCs without altering the epigenetic status of glial genes [26, 27].

**Figure 2:**
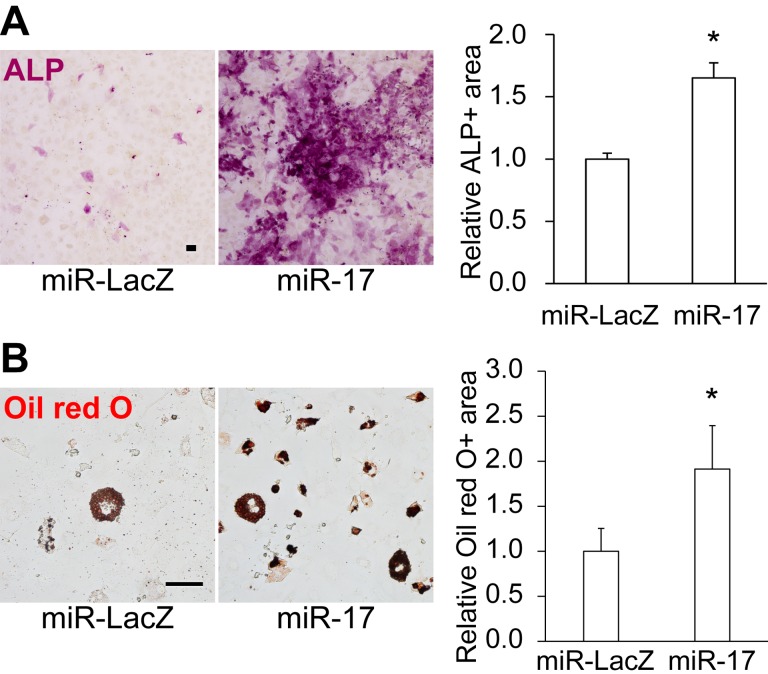
Overexpression of miR-17 restores the differentiation potential of old MSCs Overexpression of miR-17 restored the (**A**) osteogenic and (**B**) adipogenic potential of old MSCs (n = 3). Scale bars: 100 μm in **A**, 50 μm in **B**. Results are expressed as means ± SEM. *p < 0.05.

Although miR-17 is known to exert both pro- and anti-osteogenic effects on osteoblast differentiation of MSCs [28-30], the role of miR-17/106 in regulating the differentiation potential and secretory phenotype of undifferentiated MSCs remains unclear. To address this issue, we transduced old MSCs with miR-17 lentivirus, which integrates the transgene into the genome of the infected cell and continuously overexpresses EGFP and miR-17 under the control of the human elongation factor-1α promoter, and cultured them for 1 week, and retrospectively evaluated differentiation potential by measuring the area that stained positive for alkaline phosphatase (ALP) in osteoblasts and Oil red O in adipocytes. In cells overexpressing miR-17, osteogenic and adipogenic potential was significantly restored (Fig. 2A–B).

### Transplantation of miR-17-overexpressing old MSCs reverses the age-related decline in lymphopoiesis

**Figure 3:**
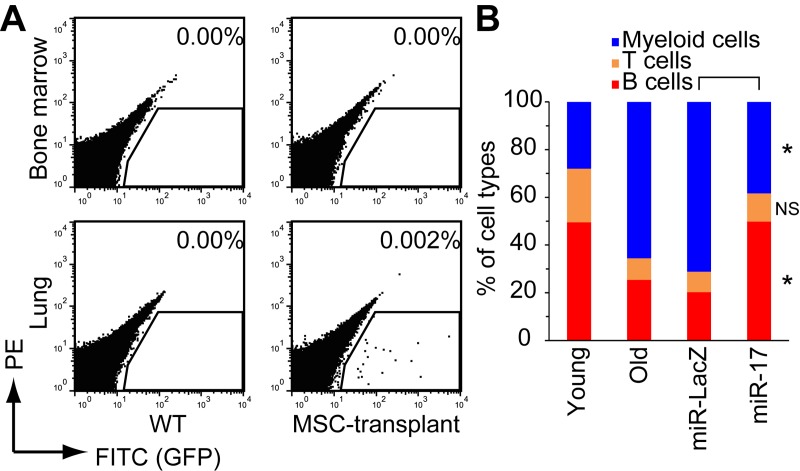
Restoration of lymphopoiesis by transplantation of miR-17-expressing old MSCs (**A**) EGFP+ transplanted cells engrafted in the lungs, but not in bone marrow. (**B**) Percentages of PB cell types were analyzed by FACS 16 weeks after transplantation (n = 4). NS, p > 0.05; *p < 0.05.

To determine whether miR-17 overexpression could restore the regenerative efficacy of old MSCs *in vivo*, we intravenously injected miR-17-overexpressing old MSCs into old mice. The transplanted GFP+ cells were detected in the lung, but not in BM (Fig. 3A and Supplementary Fig. 2), consistent with previous studies showing that most intravenously injected cultured MSCs engraft in this organ [22, 31]. Transplantation of miR-17-overexpressing, but not control miR-LacZ-expressing, old MSCs reversed the age-dependent reduction in lymphocyte number in peripheral blood (PB) (Fig. 3B and Supplementary Fig. 3). Because we detected no GFP+ transplanted cells in BM, we hypothesized that lymphopoiesis was rescued by a secretory factor derived from the transplanted MSCs.

### Gdf6 restores the osteogenic potential of old MSCs

**Figure 4:**
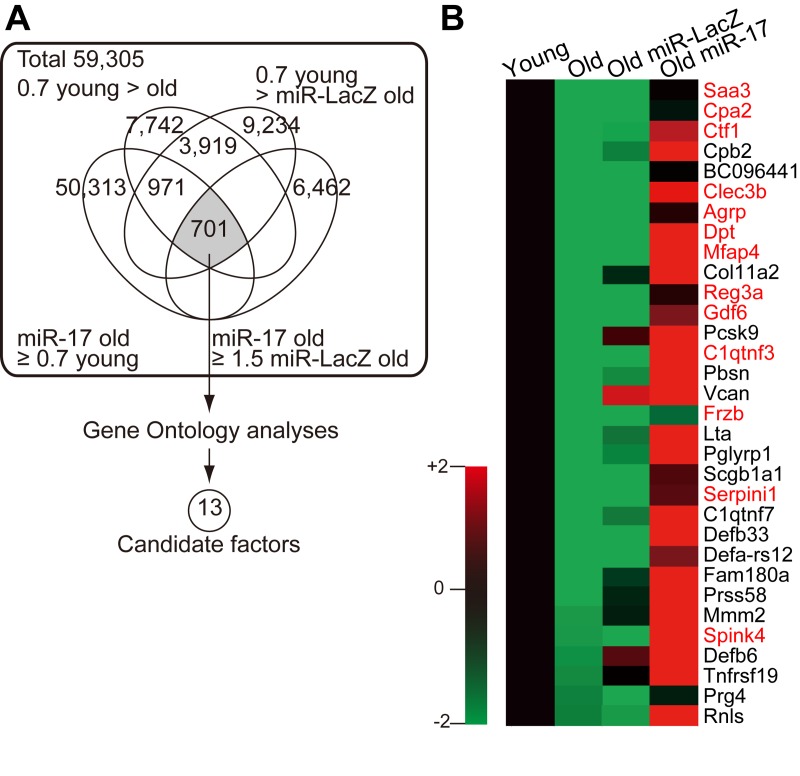
Age- and miR-17-dependent expression of MSC-secreted factors (**A**) Schematic describing the strategy for identifying candidate factors based on microarray data. (**B**) Heatmap of the expression levels of secretory factors downregulated in old MSCs. The 13 candidate factors are shown in red.

To identify the MSC-derived secretory factors responsible for restoring lymphopoiesis, we used microarrays to compare the gene expression profiles of young, old, and miR-17-overexpressing old MSCs. Genes whose expression was downregulated in old MSCs but restored by miR-17 overexpression were subjected to Gene Ontology analysis, revealing 13 candidate secretory factors (Fig. 4, Supplementary Table 4, and also see Methods).

**Figure 5:**
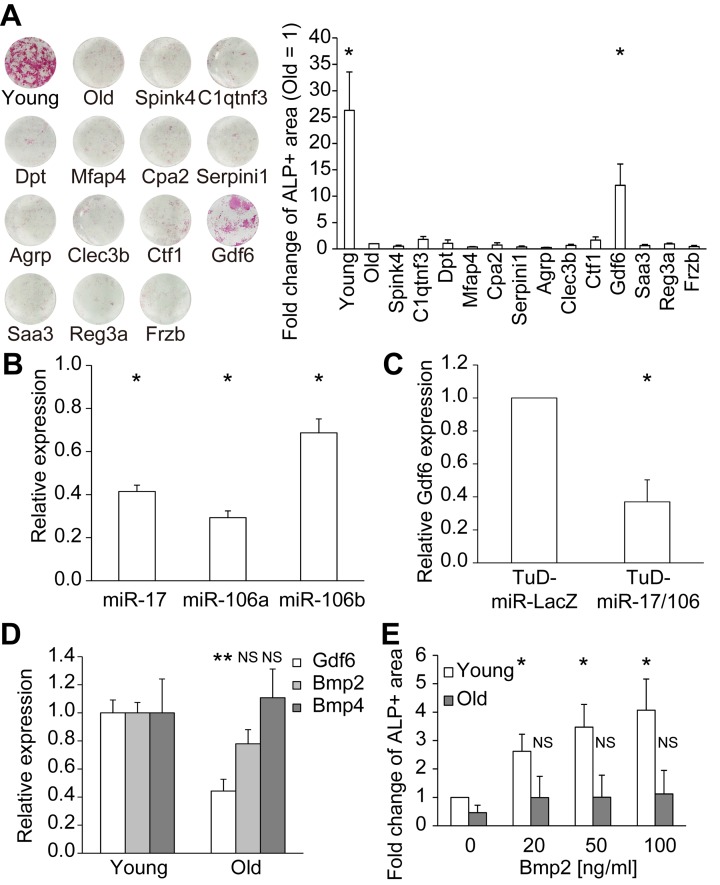
Identification of Gdf6 as an osteogenic factor derived from young MSCs (**A**) Representative images of ALP staining of differentiated MSCs. ALP activity was quantified as the percentage of ALP+ area and compared with that of old MSCs (n = 4). (**B**) TuD-miR-17/106 repressed miR-17/106 (n = 3). (**C**) Repression of miR-17/106 induced downregulation of Gdf6 (n = 3). (**D**) qPCR of Gdf6 and Bmp2/4 in young and old MSCs (n = 8). (**E**) Administration of recombinant Bmp2 promoted osteogenic differentiation in young but not old MSCs (n = 4). Results are expressed as means ± SEM. NS, p > 0.05; *p < 0.05.

We functionally analyzed these 13 candidate factors in MSC osteoblast differentiation assays. For this purpose, each factor was expressed in 293T cells, and the conditioned medium (CM) was collected. Differentiation of old MSCs into osteoblasts was induced by osteogenic differentiation media supplemented with or without 50% CM. Among the 13 candidate factors, only Gdf6 promoted osteogenic differentiation in old MSCs (Fig. 5A). Next, we investigated the dependency of Gdf6 expression on miR-17. To this end, we utilized RNA decoys, referred to as “tough decoy” RNAs (TuDs), to efficiently and stably suppress the activity of miR-17 and its paralogs miR-106a/b (Fig. 5B) [26, 32]. Repression of miR-17/106 induced downregulation of Gdf6, suggesting that expression of Gdf6 is regulated by miR-17 in MSCs (Fig. 5C).

Gdf6, which belongs to the TGF-β superfamily, is required for proper formation of eyes [33], skull [34], and certain bones and joints in the limbs [35]. Because Gdf6 and Bmp2/4 both bind to the same BMP receptors (ALK3/6 and BMPRII/ActRIIA) and signal through Smad1/5/8 [36], we compared the expression levels of Gdf6 and Bmp2/4 in young and old MSCs. Expression of Gdf6 was downregulated in old MSCs, in concordance with the diminished osteogenic potential of these cells, whereas Bmp2/4 expression remained unchanged over the course of MSC aging (Fig. 5D). Moreover, recombinant human BMP2 promoted osteogenic differentiation in a dose-dependent manner only in young MSCs, whereas BMP2-mediated osteogenic competence was suppressed in old MSCs (Fig. 5E). We confirmed that the expression levels of BMP receptors did not differ between young and old MSCs. They were approximately stable after supplementation with GDF6, BMP2, and a combination of both (Supplementary Fig. 4). There is a report about the divergent activities of BMP2 and Gdf6, despite them having similar biochemical receptor-binding affinities [37]. These results suggest that Gdf6 enhances the osteogenic potential of old MSCs independently of BMP2/4.

### Upregulation of Gdf6 ameliorates geriatric pathologies *in vivo*

**Figure 6:**
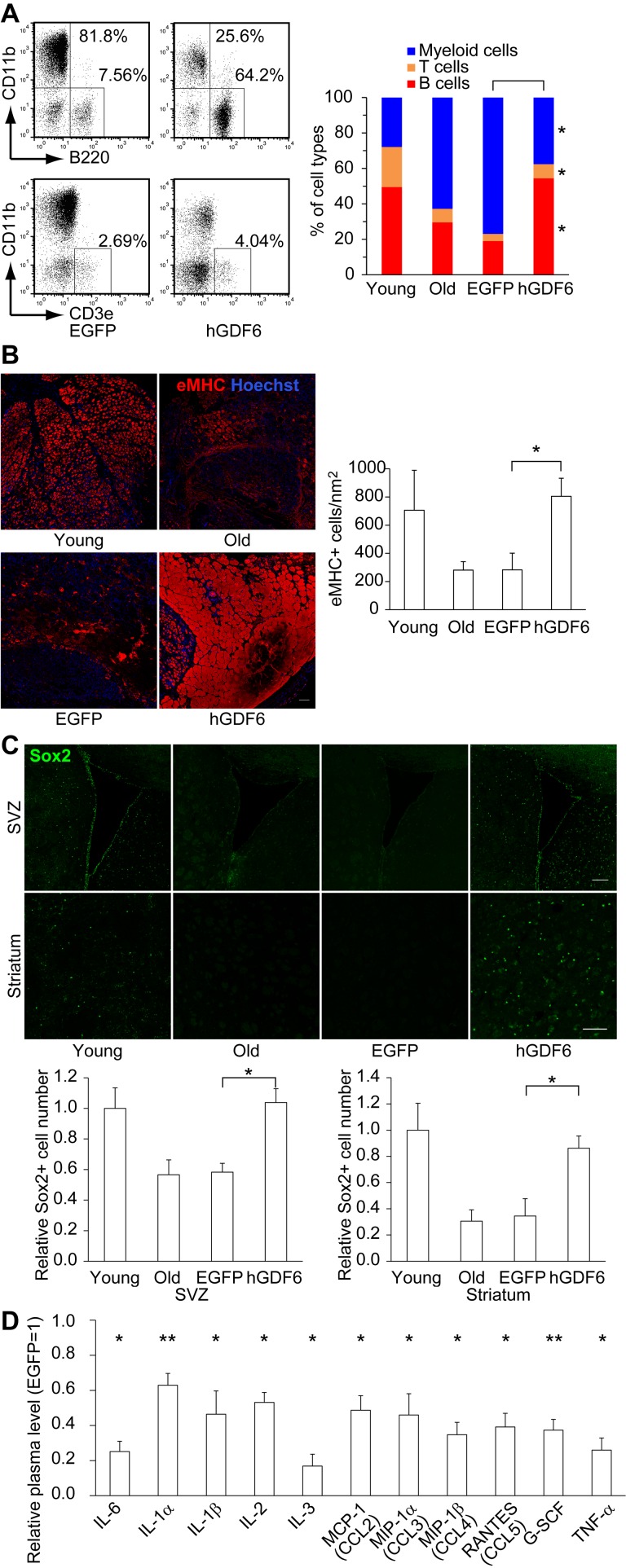
Lentiviral transduction of Gdf6 ameliorated age-related pathologies (**A**) Representative FACS plots showing the percentages of B- (B220+), T- (CD3e+), and myeloid (CD11b+) cells in PB 16 weeks after transduction. (**B**) Embryonic myosin heavy chain+ (eMHC+) regenerating myoblasts and myotubes 5 days after CTX injury were examined by immunohistochemistry. Scale bar: 100 μm. (**C**) The number of Sox2+ neural progenitor cells was elevated in hGDF6-transduced mouse brains. Scale bars: 100 μm. (**D**) Relative plasma levels of inflammatory factors (n ≥ 4). Results are expressed as means ± SEM. *p < 0.05, **p < 0.01.

Although the results presented above clearly demonstrate that Gdf6 was able to restore the differentiation potential of old MSCs, it remained unclear whether Gdf6 was the critical secretory factor responsible for restoring lymphopoiesis. To clarify this issue, we overexpressed human GDF6 (hGDF6) in 20-month-old mice by intraperitoneal lentivirus injection, and then examined the percentage of each blood cell type in PB. This method proved useful for investigating the long-term *in vivo* effects of hGDF6. We confirmed that peritoneal cells and subcutaneous adipose tissue, but not PB cells, were mainly infected with lentivirus (Supplementary Fig. 5). These lentivirus-infected cells secreted hGDF6 into blood and upregulated the plasma level at least over 16 weeks (Supplementary Fig. 6). The plasma level of Gdf6 increased with age and was further elevated by overexpression of hGDF6 (Supplementary Fig. 7). As in the MSC transplantation experiments, hGDF6 overexpression restored B- and T-cell populations to the levels observed in young mice (Fig. 6A and Supplementary Fig. 8).

In addition, we examined the influence of hGDF6 overexpression on other geriatric pathologies. To investigate the effect on muscle repair capacity, we injured the tibialis anterior muscle by focal cardiotoxin (CTX) injection 5 days before sacrifice, and then monitored the formation of embryonic myosin heavy chain-positive (eMHC+) myoblasts. In addition to mononuclear myoblasts, large numbers of eMHC+ myotubes were observed in hGDF6-overexpressing mice, as demonstrated by an increase in the eMHC+ area (Fig. 6B and Supplementary Fig. 9A). These results suggest that continuous exposure to high doses of hGDF6 accelerates muscle regeneration. Moreover, in hGDF6-overexpressing old mice, Sox2-positive (Sox2+) neural progenitors were distributed in a wide brain region that included the subventricular zone (SVZ) and striatum concomitant with improvements in the cerebrovascular network; by contrast, relatively few Sox2+ cells were detected in the brains of old control mice (Fig. 6C and Supplementary Figs. 9B–C, 10). The number of Dcx+ neuroblasts was also increased in hGDF6-overexpressing old mouse brain (Supplementary Fig. 11). We also observed broad expression of Gdf6 both in young and old mouse brains, whereas little is known about its physiological functions in the brain (Supplementary Fig. 12). Furthermore, the plasma levels of various inflammatory factors were reduced in hGDF6-overexpressing mice (Fig. 6D). Taken together, these results indicate that Gdf6 is a regenerative factor secreted from MSCs.

## DISCUSSION

Studies of age-dependent changes in secretory profiles have focused on the upregulation of diverse inflammatory factors, collectively termed the SASP. However, many secretory factors, including homeostatic growth factors, are downregulated with age. In this study, we discovered that old MSCs also exhibit age-related changes in secretory profiles, and identified Gdf6 as a regenerative factor whose expression declines with age. These results suggest that both up- and downregulation of secretory factors contribute to the development of age-related cellular dysfunction.

The major inducer of SASP is the NF-κB signaling pathway, which is activated by both genotoxic and environmental stress [38]. However, the regulatory mechanisms underlying the induction of the SASP remain largely obscure. Recently, GATA4 was identified as a novel activator of NF-κ B [39]. Selective autophagy of GATA4 is inhibited by the DNA damage response, leading to NF-κ B-mediated activation of various inflammatory factors. Likewise, it remains unclear how homeostatic factors are downregulated during senescence. Here, we showed that a subset of secretory factors are regulated by miR-17, which is also downregulated with age, suggesting that changes in the expression levels of certain miRNAs play important roles in age-related changes in secretory profiles, and also that modulation of miRNA expression enables control of the SASP.

GDF11, another member of the TGF-β superfamily, was recently identified as a regenerative factor that ameliorates age-related cardiac hypertrophy [40], slows vascular deterioration, and promotes brain neurogenesis [41] and skeletal muscle repair [42]. Despite the fact that the two factors signal via different BMP receptors and SMAD pathways [43], the regenerative effects of GDF6 observed in this study were similar to those reported previously for GDF11. Elucidation of the mechanisms underlying their effects on geriatric disorders in various tissues should improve our understanding of the aging at the organismal level.

Mutations in the GDF6 gene lead to the Klippel-Feil syndrome [44], microphthalmia, anophthalmia [33], and age-related macular degeneration (AMD) [45]. Formation of aberrant vasculature induced by GDF6 mutations has been proposed as a cause of AMD and anomalies in ocular development [33, 45, 46]. The promotion of neurogenesis and neuronal activity induced by administration of GDF11 is presumably mediated by an increase in blood flow in the brain [41]. Consistent with this, we observed improvements in the cerebrovascular network in hGDF6- overexpressing mice. These observations suggest that regeneration of vascular networks is one mechanism underlying the regenerative effects of GDF6 and GDF11 in various tissues. Smad1/5-mediated signaling functions as an activator of angiogenesis, whereas Smad2/3 signaling has a repressor effect [47], indicating that GDF6 is more suitable than GDF11 for regeneration mediated by vascular remodeling. Indeed, GDF11 can also induce the opposite effect. The active form of the GDF11 protein, which is a TGF-β family member, is processed to form biologically active carboxy-terminal dimers whose levels increase with age, inhibiting muscle regeneration [48]. Similarly, Smad1/5 signaling can inhibit endothelial cell functions [49, 50]. Moreover, context-dependent modifications of BMP/TGF-β signals by diverse cofactors further complicate the pleiotropic functions of these proteins [51-53].

Chronic inflammation accelerates systemic aging [54]. In this study, we found that plasma levels of various inflammatory factors were downregulated in the hGDF6-overexpressing old mice. Attenuation of chronic inflammation might reduce the severity of geriatric disorders or prevent them altogether. Therefore, an understanding of the mechanisms underlying the regenerative effects of GDF6 in various tissues could lead to the development of novel therapeutic agents for the treatment of age-related disorders.

## METHODS

### Cell preparation and culture

Primary MSCs (CD45−, CD31−, TER119−, Pdgfra+, and Sca-1+) were isolated from young (2–3 months) and old (> 18 months) mice and cultured as previously described [24, 25], except that the ilium was used in addition to the femur and tibia. Functional screening of the 13 secretory factors was performed using conventional BM-derived adherent stromal cells without cell sorting.

### MSC differentiation assay

Induction of osteoblast and adipocyte differentiation was performed using the Human Mesenchymal Stem Cell Osteogenic/Adipogenic Differentiation BulletKit Medium (Lonza). Differentiated osteoblasts were stained with a Leukocyte Alkaline Phosphatase Kit (Sigma-Aldrich), and adipocyte-produced fat droplets were detected by Oil red O staining. The rate of differentiation of MSCs was evaluated using the Hybrid Cell Count software of the BZ-X700 microscope (KEYENCE) and ImageJ (http://imagej.nih.gov/ij/).

### Lentivirus preparation

Lentiviruses were produced by transient transfection of human embryonic kidney 293T cells with lentiviral constructs (provided by Dr. H. Miyoshi, Keio University, and RIKEN BRC) containing the gene of interest. Sequences of TuDs were described in a previous study [26].

### Mice

Young (2–3 months) and old (> 18 months) C57BL/6JJcl mice were used. Animal care and experiments were performed according to the guidelines of the Experimental Animal Care Committee of the Yokohama Safety Center of RIKEN.

### Multiplex cytokine analysis

Plasma levels of cytokines were quantitated using the Luminex system (Bio-Rad).

### Microarray and miRNA qPCR array

Total RNA was purified from primary MSCs or MSCs cultured for infection with lentiviruses. Gene and miRNA expression profiles were investigated using microarrays (Agilent, SurePrint G3 Mouse GE microarray) and miRNA qPCR arrays (Thermo Fisher Scientific Applied Biosystems, TaqMan Array Rodent MicroRNA A+B Cards Set v3.0).

### Transplantation of MSCs

MSCs isolated from young and old mice were lentivirally transduced and intravenously transplanted into old mice as previously described [15]. The percentages of PB cell types (B220+ B cells, CD3ε + T cells, and CD11b+ myeloid cells) were analyzed by FACS using specific antibodies (BioLegend). After sacrifice, cells were prepared from BM, lungs, liver, and spleen using dispase/collagenase solution, and then analyzed by FACS to monitor MSC engraftment.

### Identification of Gdf6

Of the 59,305 probes screened by the microarray analyses, the expression levels of 701 matched four criteria: (1) <0.7-fold lower in old than in young MSCs, (2) <0.7-fold lower in miR-LacZ-overexpressing old than in young MSCs, (3) >0.7-fold higher in miR-17-expressing old than in young MSCs, and (4) >1.5-fold higher in miR-17-expressing old than in miR-LacZ-overexpressing old MSCs. These candidate genes were then further restricted to those encoding secreted proteins using Gene Ontology annotation in the DAVID online software (http://david.abcc.ncifcrf.gov), decreasing the number of candidates to 17. Thirteen of these candidate genes could be cloned from a cDNA library prepared from young MSCs and validated as miR-17-dependent, young MSC-derived secretory factors. All 13 factors were functionally evaluated by MSC osteoblast differentiation assay with CM from 293T cells expressing each factor.

### *In vivo* upregulation of hGDF6

Old mice were transduced with hGDF6 lentivirus by intraperitoneal injection (3×) every other day. Plasma levels of hGDF6 were investigated by western blotting with anti-GDF6 antibody (Sigma-Aldrich, PRS4691). To analyze the precursor form of hGDF6, blood plasma was purified using the Aurum Serum Protein Mini Kit (Bio-Rad, 7326701). Sustained upregulation of plasma levels of the active form of hGDF6 was confirmed for at least 16 weeks (Supplementary Fig. 3).

### Muscle injury

Injury was induced by focal injection of CTX in the left tibialis anterior muscle, as previously described [55]. Five days after injury, samples were obtained from the lower leg, and paraffin-embedded tissue sections were prepared for immunohistochemistry.

### Immunostaining

Immunohistochemistry was performed on 2 μm paraffin-embedded histological sections using antibodies against eMHC (MYH3; 1:50, Santa Cruz Biotechnology sc-53091) [56] and Sox2 (1:100, Abcam ab92494). Immunostained histological sections were quantitatively evaluated using the Hybrid Cell Count program of the BZ-X700 microscope (KEYENCE; Supplementary Fig. 5).

### Statistical analyses

At least three independent experiments were included in each statistical analysis. Statistical significance was determined by t-test. P-values <0.05 were considered statistically significant.

## SUPPLEMENTARY DATA TABLES AND FIGURES










