# Supplementary material for: Growth differentiation factor 6 derived from mesenchymal stem/stromal cells reduces age-related functional deterioration in multiple tissues
Source: Aging (Albany NY). 2016 Jun 14;8(6):1259–69. doi: 10.18632/aging.100982 (PMC4931831; doi:10.18632/aging.100982)
Supplement: Supplementary file 1 [file aging-08-1259-s001.pdf]

SUPPLEMENTAL DATA

Please browse Full Text version to see the links to Supplemental Tables:

Supplementary Table 1. Microarray data of young, old, miR-LacZ-overexpressing old, and miR-17-overexpressing old MSCs.

Supplementary Table 2. miRNA qPCR array data of young and old MSCs. The expression level of U6 snoRNA was used as an endogenous control.

Supplementary Table 3. Expression levels of miR-17 family members in young and old MSCs selected from Supplementary Table 2.

Supplementary Table 4. Relative expression levels of 13 candidate factors selected from Supplementary Table 1.

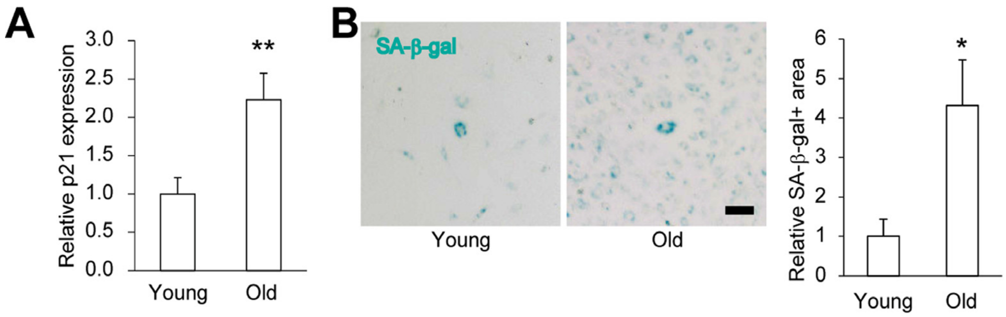

Supplementary Figure 1. Upregulation of senescence markers in old MSCs. (**A**) qPCR of p21 in young and old MSCs (n ≥ 5). (**B**) SA-β–gal activity was upregulated in old MSCs (n ≥ 4). Scale bar: 100 μm. Results are expressed as means ± SEM. *p < 0.05; **p < 0.01.

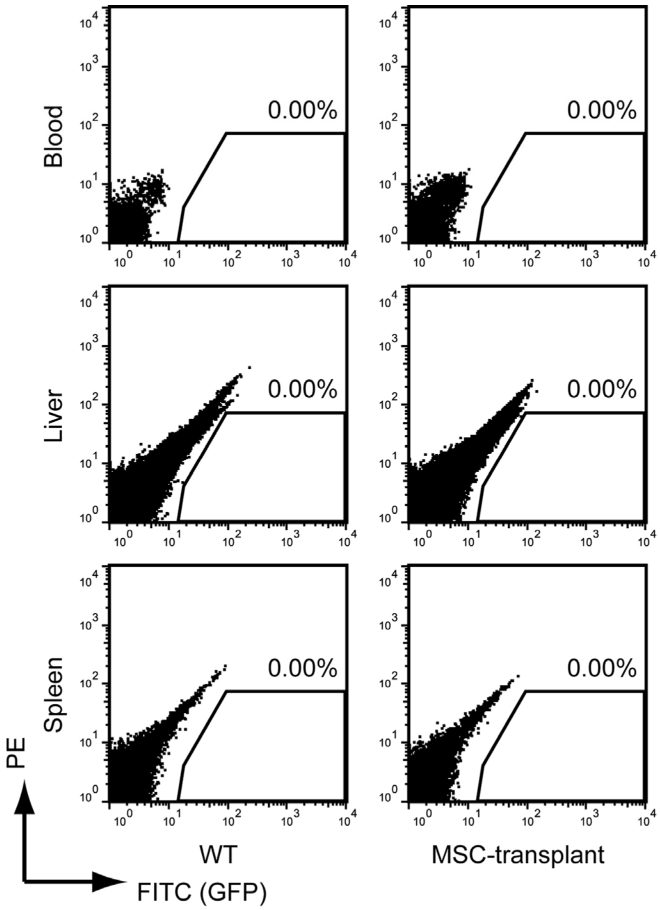

Supplementary Figure 2. FACS analyses of engraftment of transplanted GFP+ MSCs in various tissues. GFP+ lentivirus-transduced MSCs were detected only in the lungs (Fig. 3A).

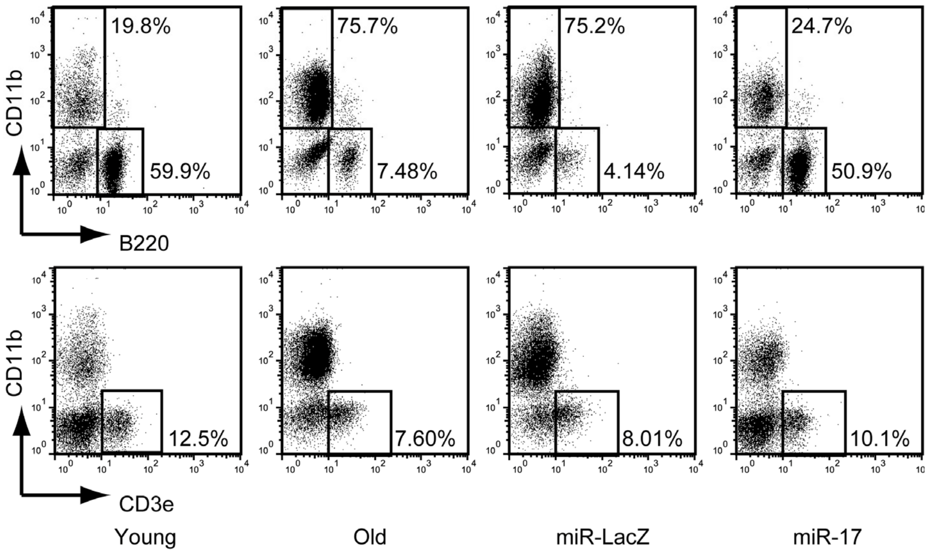

Supplementary Figure 3. Representative FACS plots of PB cells from young, old, miR-LacZ-, and miR-17-expressing MSC-transplanted mice. The decline in lymphopoiesis was reversed in miR-17-expressing MSC-transplanted mice.

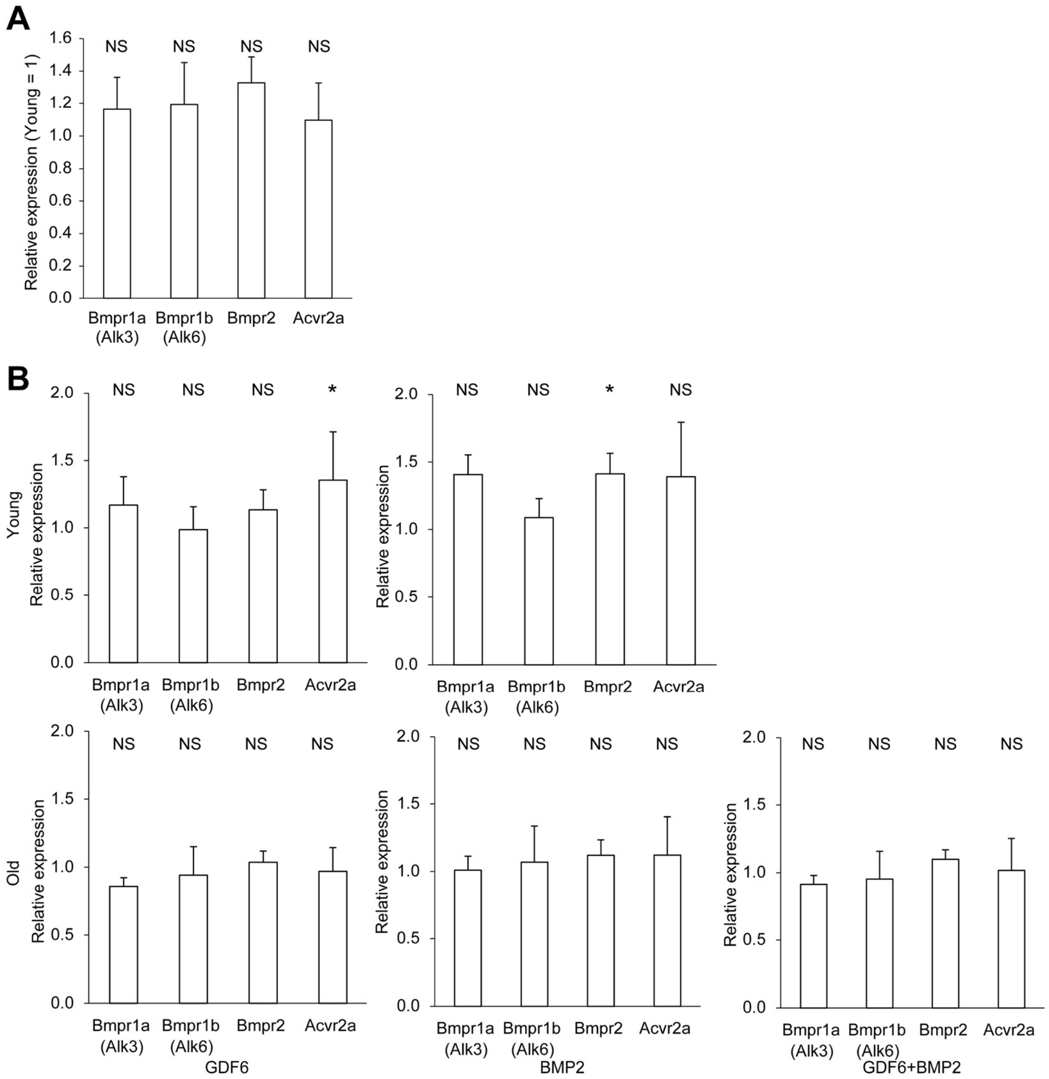

Supplementary Figure 4. Expression levels of BMP receptors. (**A**) Expression levels of BMP receptors did not differ between young and old MSCs (n ≥ 10). (**B**) Expression levels of BMP receptors were approximately stable after administration of Gdf6, BMP2, and a combination of both (n ≥ 3). Results are expressed as means ± SEM. NS, $p > 0.05$; *$p < 0.05$.

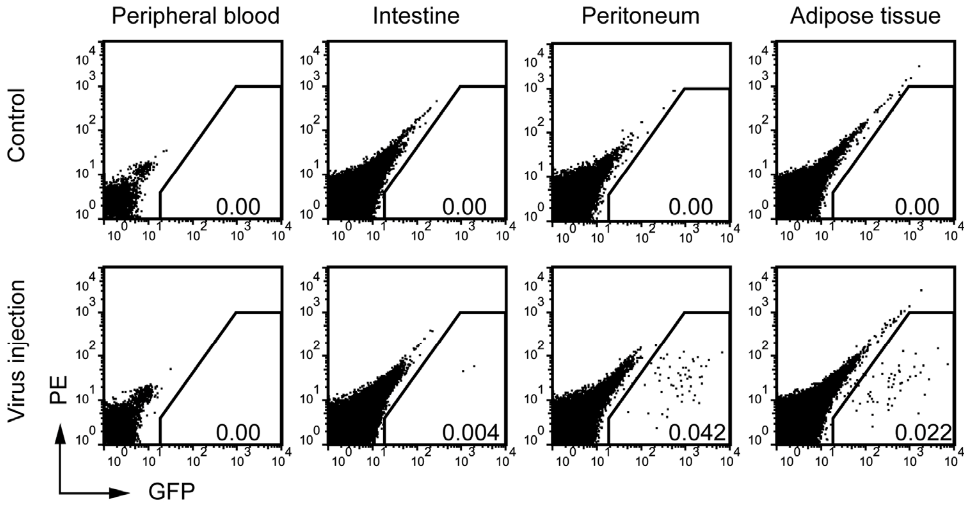

Supplementary Figure 5. Lentivirus-infected organs by intraperitoneal injection. Representative FACS plots of PB cells, intestinal cells, peritoneal cells, and subcutaneous adipose tissue cells (n ≥ 5).

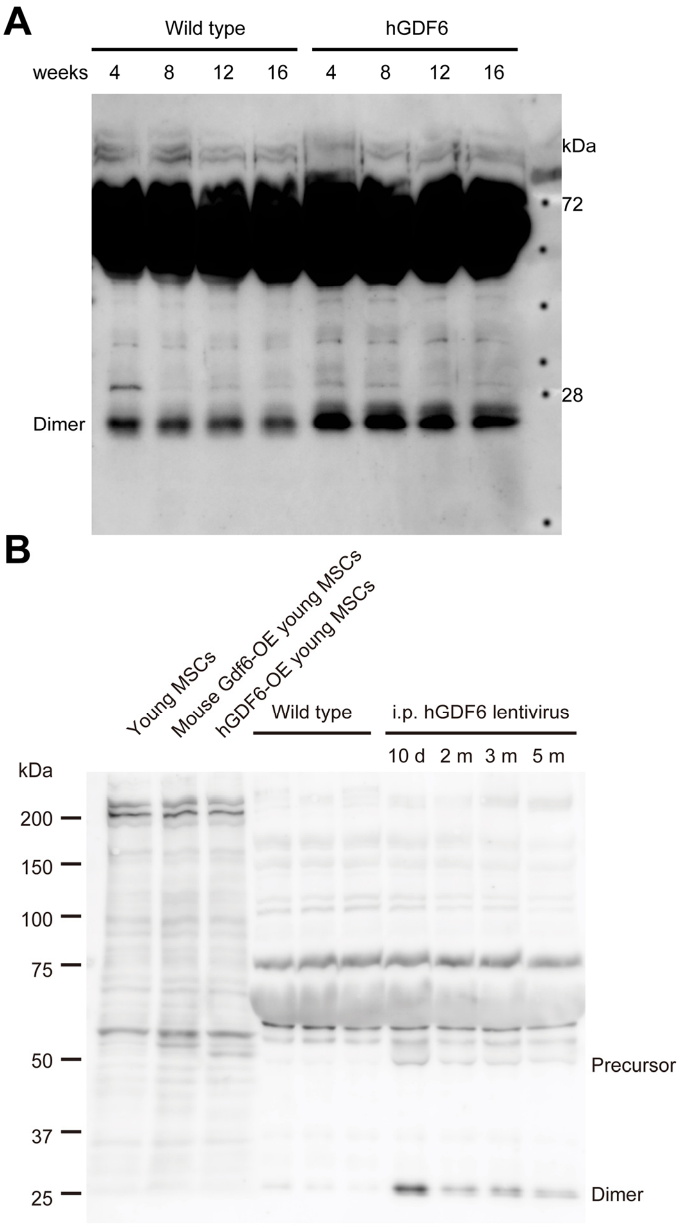

Supplementary Figure 6. Upregulation of plasma levels of hGDF6 by intravenous injection of lentivirus. Mice were transduced with hGDF6 lentivirus by three intravenous injections every other day. Upregulation of plasma levels of the activated form (**A**, **B**; Dimer) and full-length precursor (**B**; Precursor) of hGDF6 was observed for at least 16 weeks by western blot analyses.

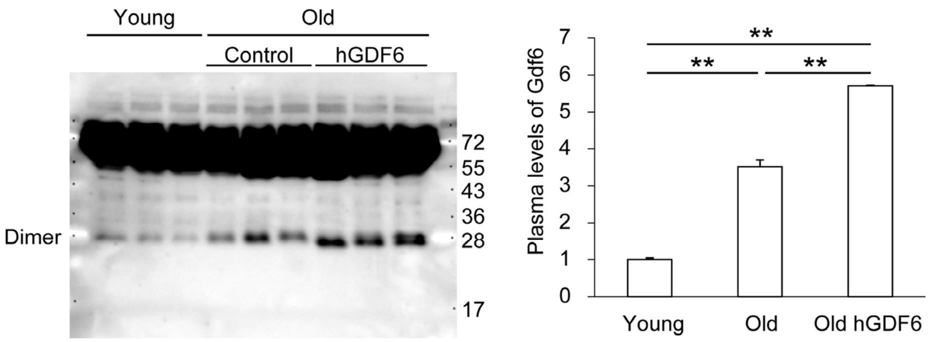

Supplementary Figure 7. Plasma levels of endogenous Gdf6. Plasma levels of the endogenous active form of Gdf6 increased with age and were further elevated by overexpression of hGDF6 (n = 3). Results are expressed as means ± SEM. **p < 0.01.

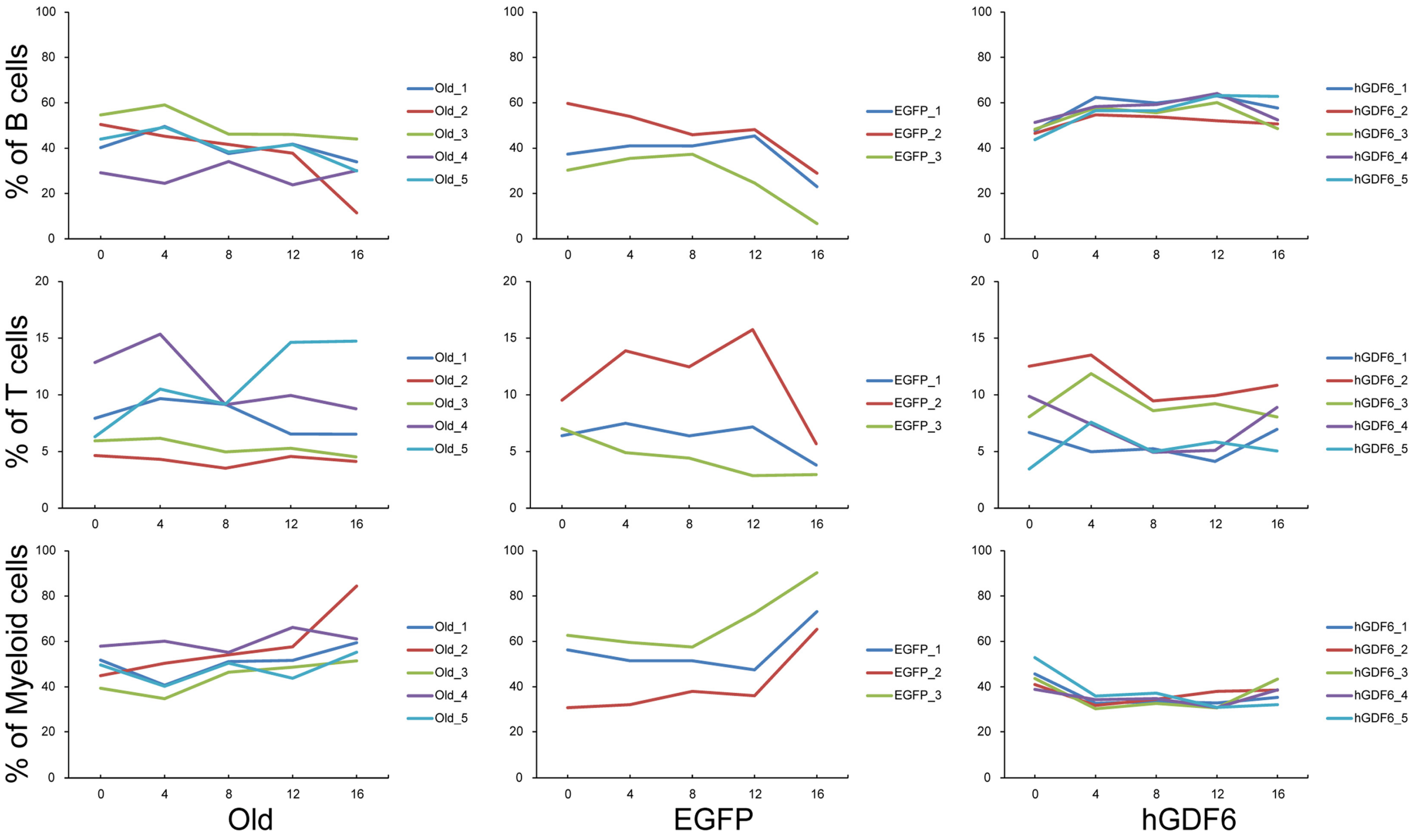

Supplementary Figure 8. Upregulation of plasma levels of hGDF6 restores lymphopoiesis. Lines show PB cell-type kinetics in old control, EGFP-, and hGDF6-upregulated mice 0, 4, 8, 12, and 16 weeks after lentiviral transduction.

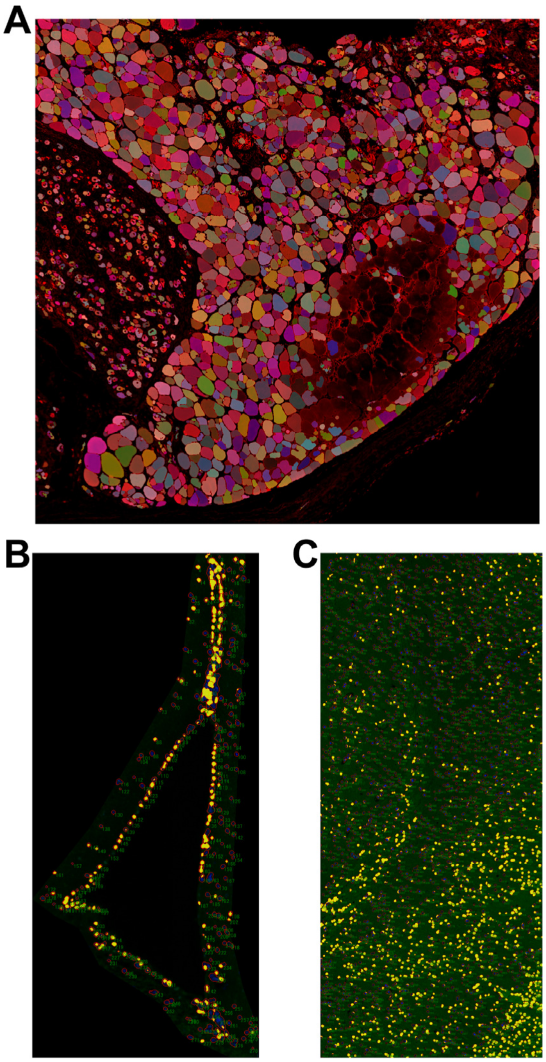

Supplementary Figure 9. Representative images of quantitation using Hybrid Cell Count. *In vivo* effects of hGDF6 on geriatric disorders were evaluated by immunohistochemistry. eMHC+ areas and Sox2+ cell numbers were quantitated using the Hybrid Cell Count software of the BZ-X700 microscope. Representative images show positive cells in (**A**) muscle (various colors), (**B**) subventricular zone (yellow), and (**C**) striatum (yellow) of hGDF6-upregulated old mice.

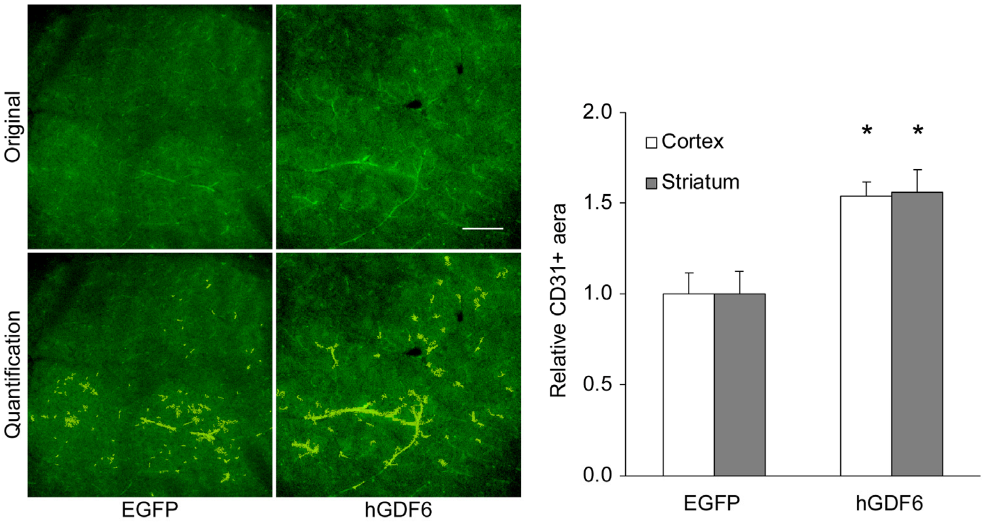

Supplementary Figure 10. Representative images of improvements in the cerebrovascular network induced by hGDF6 overexpression. Cerebral blood vessels (green) were stained with anti-CD31 antibody (BioLegend) ($n \geq 5$). Scale bar: 100 μm. Results are expressed as means ± SEM. *$p < 0.05$.

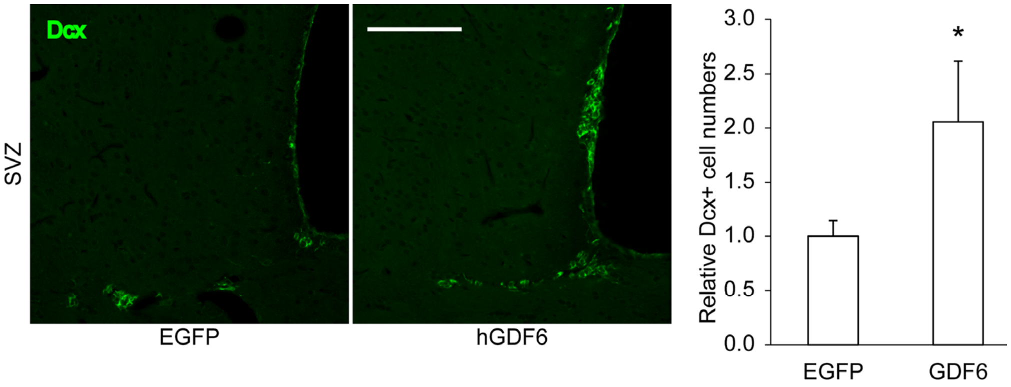

Supplementary Figure 11. The number of Dcx+ neuroblasts is increased by hGDF6 overexpression. Dcx+ neuroblasts (green) were stained with anti-Dcx antibody (Abcam) ($n \geq 5$). Scale bar: 100 μm. Results are expressed as means ± SEM. *$p < 0.05$.

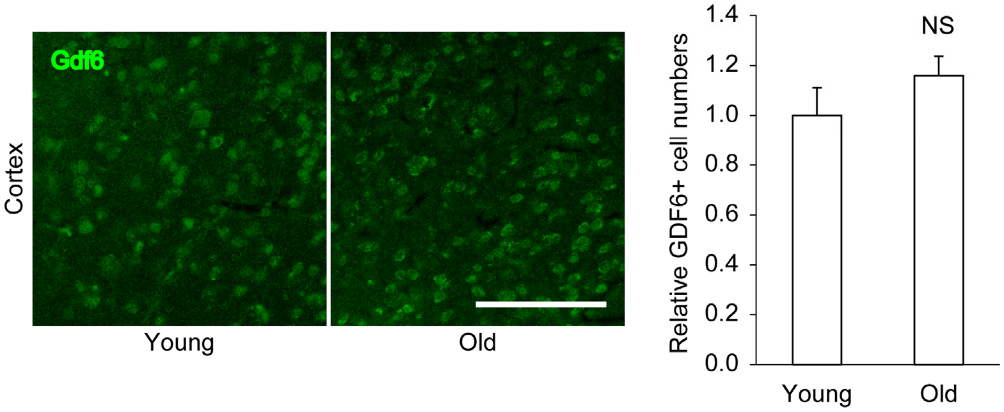

Supplementary Figure 12. Gdf6 is expressed in young and old mouse brains. Many Gdf6+ cells (green) were observed by immunohistochemical analyses of young and old mouse cortex with an anti-GDF6 antibody (Sigma) ($n \geq 5$). Scale bar: 100 μm. Results are expressed as means ± SEM. NS, $p > 0.05$.
